# First documentation of major Vip3Aa resistance alleles in field populations of *Helicoverpa zea* (Boddie) (Lepidoptera: Noctuidae) in Texas, USA

**DOI:** 10.1038/s41598-020-62748-8

**Published:** 2020-04-03

**Authors:** Fei Yang, José C. Santiago González, Nathan Little, Dominic Reisig, Gregory Payne, Rafael Ferreira Dos Santos, Juan Luis Jurat-Fuentes, Ryan Kurtz, David L. Kerns

**Affiliations:** 1 Department of Entomology, Texas A&M University, College Station, TX 77843-2475 USA; 2 Southern Insect Management Research Unit, USDA-ARS, Stoneville, MS 38776 USA; 3 Department of Entomology and Plant Pathology, North Carolina State University, Vernon G. James Research and Extension Center, 207 Research Station Road, Plymouth, NC 27962 USA; 4 Department of Biology, State University of West Georgia, Carrolton, GA 30118 USA; 5 Department of Entomology and Plant Pathology, University of Tennessee, Knoxville, TN 37996-4560 USA; 6 Cotton Incorporated, 6399 Weston Parkway, Cary, NC 27513 USA

**Keywords:** Entomology, Agricultural genetics

## Abstract

The corn earworm, *Helicoverpa zea*, is a major target pest of the insecticidal Vip3Aa protein used in pyramided transgenic Bt corn and cotton with Cry1 and Cry2 proteins in the U.S. The widespread resistance to Cry1 and Cry2 proteins in *H. zea* will challenge the long-term efficacy of Vip3Aa technology. Determining the frequency of resistant alleles to Vip3Aa in field populations of *H. zea* is critically important for resistance management. Here, we provided the first F_2_ screen study to estimate the resistance allele frequency for Vip3Aa in *H. zea* populations in Texas, U.S. In 2019, 128 *H. zea* neonates per isofamily for a total of 114 F_2_ families were screened with a diagnostic concentration of 3.0 μg/cm^2^ of Vip3Aa39 protein in diet-overlay bioassays. The F_2_ screen detected two families carrying a major Vip3Aa resistance allele. The estimated frequency of major resistance alleles against Vip3Aa39 in *H. zea* in Texas from this study was 0.0065 with a 95% CI of 0.0014–0.0157. A Vip3Aa-resistant strain (RR) derived from the F_2_ screen showed a high level of resistance to Vip3Aa39 protein, with a resistance ratio of >588.0-fold relative to a susceptible population (SS) based on diet-overlay bioassays. We provide the first documentation of a major resistance allele conferring high levels of Vip3Aa resistance in a field-derived strain of *H. zea* in the U.S. Data generated from this study contribute to development of management strategies for the sustainable use of the Vip3Aa technology to control *H. zea* in the U.S.

## Introduction

Genetically engineered crops producing insecticidal Cry and Vip proteins from the bacterium *Bacillus thuringiensis* (Bt) have been planted for control of insect pests for more than two decades^1^. Field efficacy of these Bt crops has been outstanding in controlling most target species, resulting in substantial economic, environmental and social gains^2–7^. However, with large scale adoption comes intense selection pressure for development of resistance and challenges for long-term sustainability^8,9^. To date, field-evolved practical resistance to Bt crops has been globally reported in at least 21 cases^10–15^. To delay insect resistance development, an insecticide resistance management (IRM) plan based on a “high-dose refuge” strategy has been implemented in the U.S^16^. Monitoring for evolution of resistance in field populations of the target insect species is an essential component of this IRM plan to maintain sustainability of Bt crop technologies.

The corn earworm/cotton bollworm, *Helicoverpa zea* (Boddie) (Lepidoptera: Noctuidae), is a major target pest of both Bt cotton and Bt corn in North America. Control of lepidopteran pests is achieved by the adoption of corn hybrids producing combinations of Cry1Ab, Cry1F, Cry1A.105, Cry2Ab2 and Vip3Aa20 insecticidal Bt proteins, and Bt cotton varieties producing combinations of Cry1Ac, Cry1F, Cry1Ab, Cry2Ab, Cry2Ae, and Vip3Aa19^17^. Currently, field-evolved resistance of *H. zea* to Cry1 and Cry2 proteins has been widely reported in the U.S., especially in the Southern states^15,18–21^. For example, Yang *et al*.^15^ and Kaur *et al*.^21^ documented field-evolved practical resistance of *H. zea* populations in Texas and Louisiana to Cry1A.105/Cry2Ab2 corn, respectively. In 2018, Reisig *et al*.^19^ reported field-evolved practical resistance of *H. zea* to Bt cotton containing Cry1Ac/Cry1F and Cry1Ac/Cry2Ab proteins in North Carolina. Dively *et al*.^18^ documented field-evolved resistance of *H. zea* to Cry1A.105/Cry2Ab2 corn in Maryland.

The Vip3Aa protein is produced by Bt during its vegetative stage and shows very low sequence and structural homology with Cry proteins, resulting in recognition of unique binding sites in target host cells^22,23^. Studies have shown that Vip3Aa is highly effective for control of *H. zea* in the field^24,25^ and Vip3Aa is currently used in combination with Cry1/Cry2 proteins in almost all Bt corn and Bt cotton products in the U.S^17^. However, widespread field resistance to Cry1 and Cry2 proteins in *H. zea* populations in the U.S^15,18–21^. makes Vip3Aa the only remaining effective protein against this pest in commercialized Bt crops. In addition, the Vip3Aa proteins produced by transgenic Bt corn and cotton plants are >99% identical, which places strong selection pressure on *H. zea* that feed on both crops in the Southern U.S. All these factors greatly increase the risk of resistance to Vip3Aa in *H. zea*. One of the factors affecting evolution of resistance under the “high-dose refuge” IRM model is that resistance alleles must exist at low frequency in field populations (<0.001)^8^. Consequently, it is important to determine current resistance allele frequency for Vip3Aa in field *H. zea* populations so that appropriate management strategies can be developed for the sustainable use of Bt crops including the Vip3Aa technology.

Screening with an F_2_ approach has been widely used for estimating Bt resistance allele frequency in insect populations^26,27^. In *H. zea*, frequency of resistance alleles to Cry1Ac and Cry2Aa in North Carolina during 2003 was low (0.00043 and 0.00039, respectively)^28^. Further studies to estimate resistance allele frequency have been hindered by extremely low mating frequency in single pair of male and female *H. zea* moths^29–31^. Consequently, it is very difficult to directly establish enough two-parent family-lines for F_2_ screening by single-pairing of feral males and females of *H. zea*. In this work, we conducted the first F_2_ screen study using light-trapped female moths to estimate the resistance allele frequency to Vip3Aa in *H. zea* in Texas. Furthermore, we provide the first documentation of a resistance allele conferring high levels of resistance to Vip3Aa in a field-derived *H. zea* strain in the U.S. Data generated from this study greatly contributes to improving IRM practices to increase sustainability of Bt crops producing Vip3Aa for management of *H. zea* in the U.S.

## Materials and Methods

### Insect collection and establishment of two-parent families

During May-September 2019, female moths of *H. zea* were collected from light-traps in Snook, Texas. Adult females were placed individually into a 32 oz. paper container (Choice Paper Company, Brooklyn, NY) with approximately 25 g of vermiculite at the bottom and cotton gauze at the open end for oviposition. A 30-ml plastic cup containing paper towels saturated with 10% honey water solution was placed in the center of each container and the containers were placed into an insect rearing room maintained at 26 ± 1 °C, ∼60% relative humidity (RH), and a photoperiod of 16:8 h (L:D). Progeny from each female was considered as a F_1_ family. These F1 neonates were first reared on the meridic diet (Southland Product, Inc. Lake Village, AR) using 128-well bioassay trays (C-D International, Pitman, NJ). After 7 days, the larvae were individually transferred into 30-mL plastic cups containing meridic diet (WARD’S Stonefly Heliothis diet, Rochester, New York) until pupal stage. F_2_ families were then generated by sib-mating approximately 60 viable adults of each F_1_ family line in 3.8 L paper containers (Neptune Paper Products, Newark, New Jersey) with approximately 100 g of vermiculite at the bottom. A 100-ml plastic cup containing paper towels saturated with 10% honey water solution was placed in the center of each container. These containers were maintained in the insect rearing room under the same conditions as mentioned above. Neonates of F_2_ families were screened for Vip3Aa resistance as described below.

### Vip3Aa39 protein for F_2_ screen

The Vip3Aa39 protein was produced in a recombinant *Escherichia coli* strain transformed with the pET-21b plasmid (EMD Millipore) containing the full length Vip3Aa39 insecticidal protein (GenBank accession AEH31410.1), which was a generous gift from Dr. Rongmei Liu (Northeast Agricultural University of Harbin, P. R. China). Production and purification of the Vip3Aa39 protein was as described elsewhere^32^. Briefly, an overnight preculture was used to inoculate a 1 L culture of LB media containing 100 µg/ml of ampicillin, which was incubated at 37 °C and 160 rpm, Once OD_600_ reached 0.6–0.8, Vip3Aa39 production was induced by addition of 1 M isopropyl‐ β‐d‐thiogalactopyranoside (IPTG) and overnight incubation. Bacterial cells were then collected by centrifugation (15,300 × *g*, 4 °C, 8 min) and the pellet resuspended by shaking in 100 ml of lysis buffer (20 mM phosphate buffer, pH 7.4, 500 mM NaCl, 3 mg/ml lysozyme and 10 µg/ml of DNAse I). The solution was sonicated on ice for 7 cycles of 5 seconds on/off, and then incubated overnight at 37 °C with constant shaking. Cellular debris was pelleted by centrifugation as before and the supernatant was applied to an anion exchange column (HiTrap Q HP) equilibrated in 20 mM Tris-HCl (pH 9) buffer connected to an AKTA Pure chromatography system (GE Healthcare). The Vip3Aa39 protein was recovered in the flow through, and then the pH of the solution was adjusted to 4.5 to precipitate contaminant proteins, which were collected by centrifugation (15,300 × g, 4 °C, 20 min). The concentration of Vip3Aa39 in the supernatant was estimated using SDS-10%PAGE combined with densitometry using BSA as standard^33^. The Vip3Aa39 protein shows 94.93% and 94.80% homology compared to the Vip3Aa19 and Vip3Aa20 proteins, respectively.

### Screening of F_2_ neonates

Before the F_2_ screen bioassays, the toxicity of Vip3Aa39 protein was evaluated against a known susceptible population of *H. zea*. The results showed that the median lethal concentration (LC_50_) that caused 50% mortality of the susceptible population was 0.17 μg/cm^2^ with a 95% CL of 0.14–0.21 μg/cm^2^, and 100% susceptible insects were killed on a 3.0 μg/cm^2^ concentration (see Results). Therefore, we used 3.0 μg/cm^2^ Vip3Aa39 as the diagnostic concentration for the F_2_ screens. Susceptibility to Vip3Aa39 of the F_2_ families of *H. zea* was evaluated using a diet-overlay bioassay as described in Yang *et al*.^15^. Briefly, 0.8 ml of liquid diet (Southland Product, Inc. Lake Village, AR) was dispensed using repeater pipets into each well of 128-well bioassay trays (C-D International, Pitman, NJ). Once the diet cooled and solidified, repeater pipets were used to overlay 40 μl of a Vip3Aa39 protein solution suspended in 0.1% Triton-X100 onto the diet surface of each well. Once the protein solution was air-dried, one neonate (<24 h) was released on the diet surface in each well. Wells were covered with vented lids (C-D International, Pitman, NJ). For each F_2_ family, 128 neonates were screened against Vip3Aa39 protein. Larval survival and development of the F_2_ neonates were also examined on the control diet, with four replications and 32 insects per replication. The control diet was prepared by overlaying the same amount of buffer solution and 0.1% Triton-X100. The bioassay trays were maintained in an insect rearing room under the conditions of 26 ± 1 °C, 60% RH, and a 16:8 (L:D) h photoperiod. Larval survival and their instar were recorded after 7 days. In addition, larval survival and development of a susceptible strain (SS) of *H. zea* was also evaluated on both control and 3.0 μg/cm^2^ Vip3Aa39 using the same methods as described above. In each bioassay for SS, there were four replications with 32 larvae in each replication. The SS strain was originally collected from LSU AgCenter Macon Ridge Research Station in Franklin Parish in May 2016, and has been documented to be susceptible to Cry1Ac, Cry2Ab2, and Vip3Aa proteins^15^.

### Establishment of potential resistant family and confirmation test

In a total of 114 F_2_ families tested, the F_2_ screen identified five families having survivorship (≥2^nd^ instar) on the discriminatory 3.0 μg/cm^2^ Vip3Aa39 protein concentration after 7 days (Table 1). These survivors were reared on the control meridic diet and used to establish potential resistant families. Survivors from each family were first crossed with SS to create potential RS families. Due to the limited number of F_2_ survivors, only three potential RS families (LT#16, LT#70, and LT#116) were successfully established (see Results). These potential RS insects were then sib-mated and neonates from these sib-mated colonies were evaluated again using diet-overlay bioassay as described above. For each sib-mated potential RS family, 512 insects were assayed at the concentration of 3.0 μg/cm^2^ Vip3Aa39. According to Mendelian genetics, if resistance is controlled by one locus with two alleles; S (susceptible) and R (resistant), the F_2_ strains from sib-mating RS are expected to consist of 25% RR, 50% RS and 25% SS genotypes. The confirmation test showed that survivors were only derived from LT#70 (see Results), suggesting that the LT#70 family possessed major resistance alleles against Vip3Aa39 protein. Survivors of LT#70 from the confirmation test were used to establish a resistant colony (renamed as RR). To further verify if the survival of RR in the F_2_ screen was due to resistance to the Vip3Aa39 protein, susceptibility of RR, along with SS to Vip3Aa39 protein was determined using the full range dose response bioassays as described below.

**Table 1:** Families containing survivors of *Helicoverpa zea* from the F_2_ screen on 3.0 μg/cm^2^ of Vip3Aa39 protein.

Family No.	No. insects screened	No. survivor	No. insect within instar		
			2^nd^	3^rd^	4^th^
LT#14	128	1	1	0	0
LT#16	128	5	5	0	0
LT#18	128	4	3	1	0
LT#70	128	7	0	2	5
LT#116	128	4	4	0	0

### Dose response bioassays

Susceptibility to Vip3Aa39 in RR and SS strains of *H. zea* was evaluated using a diet-overlay bioassay as described in Yang *et al*.^15^. In the full range bioassay, concentrations of Vip3Aa39 ranged from 0, 0.0316, 0.1, 0.316, 1, 3.16, 10, 31.6 to100.0 μg/cm^2^. Each combination of insect population by Vip3Aa39 protein concentration was replicated four times with 16 larvae in each replication. Bioassay trays were placed in an environmental chamber maintained at 26 ± 1 °C, 60% RH, and a 16:8 (L:D) h photoperiod. Larval mortality and instar were recorded on the 7^th^ day after inoculation.

### Data analysis

In the diet bioassays, larval mortality was calculated as mortality % = 100 * (number of dead larvae + number of surviving larvae that were still in the first instar)/total number of insects assayed, and larval mortality at each concentration was corrected based on the control mortality^34^. For the dose response bioassay, probit analysis was conducted to determine the median lethal concentration (LC_50_) that caused 50% mortality and the corresponding 95% confidence limit (CL)^35^. The LC_50_ value of an insect was considered greater than the highest Bt protein concentration used in the bioassay if larval mortality was <50% at the highest concentration. Resistance ratio for RR was calculated as its LC_50_ value divided by the LC_50_ of SS. Moreover, larval mortality and instar data were analyzed using a two-way ANOVA with insect genotype and protein concentration as the two main factors^35^. To meet the normality assumptions for an ANOVA test, original data on the percentage of larval mortality and larval instar were transformed using arcsine (χ^0.5^) and log (x + 1) scale, respectively. Treatment means were separated using Tukey’s HSD test at α = 0.05 level^35^.

The estimated resistance allele frequency and its corresponding 95% confidence intervals were estimated using the method described in Andow and Alstad^27^. The probability (detection power) that a resistance allele can be detected in a family if existing was estimated according to Stodola and Andow^36^.

## Results

### Survival of F_2_ families in the F_2_ screen bioassay

After 7 days, survival of SS on control diet was 100.0 ± 0.0% with 1.6% 3^rd^ and 98.4% 4^th^ instar larvae. In contrast, there were no SS survivors on diet with 3.0 μg/cm^2^ Vip3Aa39 protein. Based on these results, the concentration of 3.0 μg/cm^2^ Vip3Aa39 as discriminating dose was sufficient to kill all susceptible *H. zea* in the F_2_ screening, and thus appropriate to detect Vip3Aa39 resistant individuals. The 7-day larval survival of the F_2_ families on control diet was 96.1 ± 1.6%, which was not significantly different (*P* > 0.05) compared to the survival of SS.

A total of 14,592 insects from 114 F_2_ families were screened using the Vip3Aa39 discriminating dose in this study. After 7 days, five F_2_ families had survivors (≥2^nd^ instar) on the 3.0 μg/cm^2^ Vip3Aa39 protein (Table 1). Three (LT#14, LT#16, and LT#116) of them contained only 2^nd^ instar larvae. The LT#18 family contained three 2^nd^ instar and one 3^rd^ instar larvae. The LT#70 family had two 3^rd^ and five 4^th^ instar larvae.

### Resistance confirmation

On the confirmation test, insects derived from sib-mating of potential RS families of LT#16 and LT#116 were all killed on 3.0 μg/cm^2^ Vip3Aa39 at the 7-day. However, insects derived from sib-mating of potential RS family of LT#70 had a survival of 24.8% on the discriminating Vip3Aa39 dose after 7 days, which was not significantly (P > 0.05) different from the expected survivorship of 25% for homozygous resistant insects according to Mendelian genetic transmission for monogenic recessive resistance. These survivors included eight 2^nd^, eight 3^rd^, and one hundred and eleven 4^th^ instar larvae, suggesting that only LT#70 family (renamed as RR) probably carries a major resistance allele conferring to Vip3Aa39 protein.

### Susceptibility of SS and RR populations of *H. zea* to Vip3Aa39 protein

**Figure 1:**
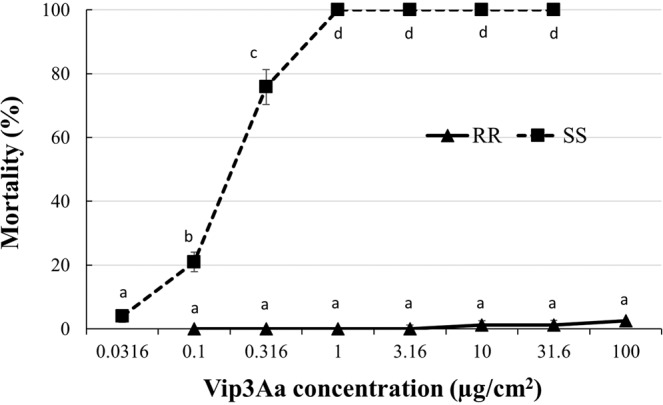
Concentration-larval mortality response of the SS and RR strains of *Helicoverpa zea* to Vip3Aa39 protein. Mean percentage mortality values followed by a different letter are significantly different (Tukey’s HSD test, α = 0.05).

Larvae of the SS strain were highly susceptible to Vip3Aa39, with 75.8% mortality observed at 0.316 μg/cm^2^ and 100% mortality at 1–31.6 μg/cm^2^ (Fig. 1). The LC_50_ value of SS against Vip3Aa39 protein was estimated as 0.17 μg/cm^2^ with a 95% CL of 0.14–0.21 μg/cm^2^ (Table 2). In contrast, larvae from the RR strain was highly resistant to Vip3Aa39 protein, and showed no differences (*P* > 0.05) in mortality (0–2.5%) across all the tested concentrations (Fig. 1). Consequently, we were unable to estimate the LC_50_ for strain RR, as the mortality at the highest tested concentration of 100.0 μg/cm^2^ was only 2.5% (Fig. 1). Based on this observation, the LC_50_ value for RR was considered >100.0 μg/cm^2^, with an estimated resistance ratio >588.0-fold relative to SS (Table 2).

**Table 2:** Mortality response (LC_50_) of different populations of *Helicoverpa zea* to Vip3Aa39 protein in diet-overlay bioassays.

Insect	N^*^	LC_50_ (95% CL) (μg/cm^2^)^#^	Slope ± SE^£^	X^2^	df	Resistance ratio^§^
SS	512	0.17 (0.14, 0.21)	2.84 ± 0.29	10.5	26	—
RR	512	>100	/	/	/	>588.0

^*^Total number of neonates assayed.

^#^Median lethal concentration (LC_50_) that caused 50% mortality and the corresponding 95% confidence limit (CL). The LC_50_ value of an insect population was considered to be greater than the highest Bt protein concentration used in the bioassay if its larval mortality was <50% at the highest concentration. Larval mortality was calculated based on the number of dead larvae plus survivors that were still in the first instar divided by the total number of insects assayed.

^£^SE, standard error.

^§^Resistance ratio was calculated using the LC_50_ value of RR divided by the LC_50_ of SS.

The main effect of insect population and protein concentration on larval mortality was significant for the Vip3Aa39 protein (*F* = 3401.95; df = 1, 42; *P* < 0.0001 for insect population and *F* = 162.93; df = 7, 42; *P* < 0.0001 for protein concentration). The effect of the interaction of insect population and protein concentration was also significant (*F* = 67.20; df = 5, 42; *P* < 0.0001).

**Figure 2:**
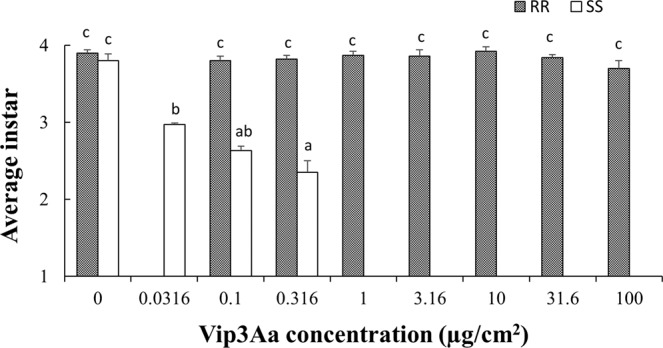
Average larval instar detected for SS and RR populations of *Helicoverpa zea* on different concentrations of Vip3Aa39 protein, as indicated. Mean values followed by a different letter are significantly different (Tukey’s HSD test, α = 0.05).

The effects of insect population, protein concentration and their interactions on larval instar were all significant for the Vip3Aa39 protein (*F* = 226.72; df = 1, 36; *P* < 0.0001 for insect population; *F* = 15.44; df = 8, 36; *P* < 0.0001 for protein concentration; and *F* = 48.74; df = 2, 36; *P* < 0.0001 for the interactions). Larval growth on control diet was similar (*P* > 0.05) between SS and RR after 7 days, with an average instar of 3.80 and 3.90, respectively (Fig. 2). Larval development of SS at 0.0316–0.316 μg/cm^2^ of Vip3Aa39 protein was significantly (*P* < 0.05) slower than that on control diet (Fig. 2). RR showed no differences (*P* > 0.05) in larval growth across all the concentrations from 0–100 μg/cm^2^ with an average instar of 3.84 (Fig. 2).

### Major resistance allele frequency for Vip3Aa39

Based on the results of F_2_ screening, resistance confirmation and dose response bioassays, two out of 114 families collected from Snook (Texas) were presumed to carry a major resistance allele against Vip3Aa39 protein. Thus, the expected resistance allele frequency of the Snook populations of *H. zea* to Vip3Aa39 protein is estimated to be 0.0065 with a 95% CI of 0.0014–0.0157. The F_2_ screen had a detection power of 98.2%.

## Discussion

The corn earworm/cotton bollworm, *H. zea*, is a major target pest of Bt corn and cotton in the U.S. Within the past four years, scientists have been reporting field-evolved resistance of *H. zea* to Cry1 and/or Cry2 proteins in Bt corn and Bt cotton^15,18–21^. This widespread occurrence of Cry1 and Cry2 resistance will challenge the long-term efficacy of Vip3Aa protein in the pyramided Bt crops against *H. zea*. In order to make scientifically-based decisions concerning Bt resistance management, accurate estimations of the frequency of Vip3Aa resistant alleles in field populations of *H. zea* are urgently needed. Prior to this study, resistance allele frequency of Vip3Aa has been estimated only for three insect species in a few locations, including *Spdoptera frugiperda* in Brazil and U.S.^37–41^, and *Helicoverpa armigera* and *Helicoverpa punctigera* in Australian^42^. For example, the estimated resistance allele frequency to Vip3Aa in *H. punctigera* and *H. armigera* was 0.008 (95% CI, 0.004–0.015) and 0.027 (95% CI, 0.019–0.038), respectively during 2009–2010 in Australia^42^. The calculated resistance allele frequency to Vip3Aa in *S. frugiperda* was 0.0009 with a 95% CI of 0–0.0021 during 2013–2015 in Brazil^37,38^. To our knowledge, few data are currently available regarding the baseline resistant allele frequency of Vip3Aa in *H. zea*. Here, we provide the first F_2_ screening study to estimate the resistance allele frequency of field populations of *H. zea* against Vip3Aa protein in Texas, U.S. Based on the results, we estimated a frequency of 0.0065 for Vip3Aa resistance alleles in this region, which can be considered as relatively low, albeit higher than the desired <0.001 frequency for efficacy of the high-dose refuge strategy. Furthermore, results from confirmation tests showed that 24.8% of insects derived from crosses of RS with RS survived well on the diagnostic concentration of Vip3Aa protein, suggesting that Vip3Aa resistance in *H. zea* appears to be mostly monogenic and recessive.

Previous studies have shown that field populations of *H. zea* could mate multiple times, but in most cases females mated only once and >66% of the females carried a single spermatophore^43,44^. In addition, independently of the number of matings, a single male will typically gain sperm precedence and genetic material contributing to offspring will be from only one male^28,44^. The traditional method used for F_2_ screens includes establishing two-parent family-lines by single-pairing of feral males and females derived from larval collection^26,39,40^. However, successful mating frequency is extremely low for *H. zea* with the traditional method. In 2017–2018, we paired hundreds of feral males and females of *H. zea*, and only obtained <10 successful matings (Unpublished observation). Studies conducted by Blanco *et al*.^31,45^ indicated that mating frequency for *H. zea* could be significantly increased when mating feral individuals with laboratory individuals. Although this modified F_2_ screen method only represents half of the genetics of field populations, our preliminary tests have shown that this strategy works efficiently to establish iso-line families of *H. zea* when the light trap method is not accessible.

There are three key factors favoring success of the high-dose refuge strategy to delay evolution of resistance: low resistance allele frequency, recessive inheritance and abundant refuges of non-Bt plants^8,46^. The data generated from this study imply that the risk of evolution of Vip3Aa resistance in *H. zea* could be low if other resistance management expectations are realized. Another two factors that could help delay insect resistance to Bt crops are fitness costs and incomplete resistance^8,9^. Using the limited survivors from the F_2_ screen, we successfully established a resistant (>588-fold) population of *H. zea* against Vip3Aa protein. This Vip3Aa-resistant population showed no differences in mortality and average instar between the control and 0.1–100 μg/cm^2^ of Vip3Aa, indicating that the Vip3Aa resistance is complete. Moreover, the bioassay data did not detect differences between the susceptible and Vip3Aa resistant populations on control diet in terms of survivorship and average instar, suggesting the lack of relevant fitness costs. It is critical to point out that this preliminary lack of observed fitness costs and complete resistance are just based on comparison between SS and RR on control diet and parameters at the individual level. However, previous studies have suggested that fitness costs of Bt resistance can be influenced by various factors, including host plant species, allelochemicals, pathogens and parameters at the individual level or population level^47–51^. Chen *et al*.^51^ found recessive fitness costs of reduced pupal weight and growth rate at the individual level in a Vip3A-resistant population of *S. frugiperda* on sorghum, but no measurable fitness costs were detected on corn, cotton, or meridic diet. In comparison, no evident fitness costs were observed at the population level (net reproductive rate and intrinsic rate of population increase) of Vip3A resistance in *S. frugiperda* on any of those hosts. Further research is needed to evaluate fitness costs and complete resistance associated with the Vip3A resistance in *H. zea* on non-Bt plants at both individual and population levels.

In a previous study, Yang *et al*.^15^ reported a high infestation of *H. zea* larvae on Leptra corn ears, which expresses Cry1Ab, Cry1F and Vip3A proteins. The subsequent diet-overlay bioassays with Vip3Aa51 protein (100% identical to Vip3Aa39) showed that *H. zea* populations collected from Cry1Ab + Cry1F + Vip3A corn had a significant resistance ratio of >20-fold relative to a susceptible population of *H. zea* collected from Cry1F + Cry1A.105 + Cry2Ab2 corn at the same location^15^. It should be noted that insects used for F_2_ screen in the present study were collected at the same location as Yang *et al*.^15^. In addition to the two out of 114 families of *H. zea* surviving with 3^rd^ instar larvae on the diagnostic Vip3Aa concentration, three additional families survived with numerous 2^nd^ instar larvae. In addition, unexpected damage and survival of *H. zea* on Vip3Aa cotton and corn were also observed in 2019 field trials at the same location (Yang *et al*., unpublished data). Moreover, a field trial conducted in Stoneville, MS in 2019 showed that 16 out of 200 randomly sampled ears of Leptra corn were damaged by *H. zea* in the field, with an average of 5.5 damaged kernels per ear. The underlying mechanisms for these unexpected survivals of *H. zea* on the Vip3Aa traits are unclear. It is possible that plants at some stages could not produce a high dose of Bt protein and larvae with minor resistance genes could be selected and would, over time, decrease the efficacy of these Bt crops. For example, Yang *et al*.^15^ reported that *H. zea* populations collected from Cry1Ab + Cry1F + Vip3A corn could survive well on the WideStrike3 (Cry1F + Cry1Ac + Vip3A) cotton leaves, with a survivorship of 41.7% compared to that of 3.3% for the susceptible population. Nevertheless, data from the present study suggest that resistance allele frequency for Vip3Aa in *H. zea* in the field is not rare (<0.0001). Resistance management strategies must be adopted to preserve the sustainable use of Vip3Aa technology, especially with the widespread acceptance of Vip3Aa technology in corn and cotton in the southern U.S. With the success of the modified F_2_ screening method, we plan to screen larger population sample sizes from multiple locations and crops to accurately estimate the current status of Vip3A resistance allele frequency for *H. zea* in the U.S. Importantly, and as far as we know, this is the first population of *H. zea* in the world showing high resistance to Vip3Aa protein. The availability of this resistant strain provides a valuable resource to obtain information for the sustainable use of Vip3Aa technology for control of *H. zea* and improve insect resistance management strategies.

## References

[1] James, C. Global status of commercialized biotech/GM crops: 2017. Brief No. 53. ISAAA: Ithaca, NY, USA. (2018).

[2] Carpenter JE (2010). Peer-reviewed surveys indicate positive impact of commercialized GM crops. Nat. Biotechnol..

[3] Edgerton MD (2012). Transgenic insect resistance traits increase corn yield and yield stability. Nat. Biotechnol..

[4] Hutchison WD (2010). Areawide suppression of European corn borer with Bt maize reaps savings to non-Bt maize growers. Science.

[5] Kathage J, Qaim M (2012). Economic impacts and impact dynamics of Bt (Bacillus thuringiensis) cotton in India. Pro. Nat. Acad. Sci. USA.

[6] Lu YH, Wu KM, Jiang YY, Guo YY, Desneux N (2012). Widespread adoption of Bt cotton and insecticide decrease promotes biocontrol services. Nature.

[7] Wu KM, Lu YH, Feng HQ, Jiang YY, Zhao JZ (2008). Suppression of cotton bollworm in multiple crops in China in areas with Bt toxin-containing cotton. Science.

[8] Gould F (1998). Sustainability of transgenic insecticidal cultivars: integrating pest genetics and ecology. Annu. Rev. Entomol..

[9] Tabashnik BE, Brévault T, Carrière Y (2013). Insect resistance to Bt crops: lessons from the first billion acres. Nat. Biotechnol..

[10] Tabashnik BE, Carrière Y (2017). Surge in insect resistance to transgenic crops and prospects for sustainability. Nat. Biotechnol..

[11] Grimi DA (2018). Field‐evolved resistance to Bt maize in sugarcane borer (Diatraea saccharalis) in Argentina. Pest. Manag. Sci..

[12] Smith JL, Lepping MD, Rule DM, Farhan Y, Schaafsma AW (2017). Evidence for field-evolved resistance of Striacosta albicosta (Lepidoptera: Noctuidae) to Cry1F Bacillus thuringiensis protein and transgenic corn hybrids in Ontario, Canada. J. Econ. Entomol..

[13] Chandrasena DI (2018). Characterization of field‐evolved resistance to Bacillus thuringiensis‐derived Cry1F δ‐endotoxin in Spodoptera frugiperda populations from Argentina. Pest. Manag. Sci..

[14] Gassmann, A. J., Shrestha, R. B., Kropf, A. L., St Clair, C. R. & Brenizer, B. D. Field‐Evolved Resistance by Western Corn Rootworm to Cry34/35Ab1 and other Bacillus thuringiensis Traits in Transgenic Maize. *Pest Manag Sci*, 10.1002/ps.5510 (2019). 10.1002/ps.5510 31207042

[15] Yang F (2019). Occurrence and ear damage of Helicoverpa zea on transgenic Bacillus thuringiensis maize in the field in Texas, US and its susceptibility to Vip3A protein. Toxins.

[16] US-EPA (US Environmental Protection Agency), Report of subpanel on Bacillus thuringiensis (Bt) plant-pesticides and resistance management. Available at, https://archive.epa.gov/scipoly/sap/meetings/web/pdf/finalfeb.pdf (1998).

[17] DiFonzo, C., Porter, P. & Tilmon, K. The Handy Bt trait table, https://lubbock.tamu.edu/files/2018/01/BtTraitTableJan2018.pdf (2018).

[18] Dively GP, Venugopal PD, Finkenbinder C (2016). Field-evolved resistance in corn earworm to Cry proteins expressed by transgenic sweet corn. PLoS One.

[19] Reisig DD (2018). Long-term empirical and observational evidence of practical Helicoverpa zea resistance to cotton with pyramided Bt toxins. J. Econ. Entomol..

[20] Bilbo TR, Reay-Jones FPF, Reisig DD, Greene JK (2019). Susceptibility of corn earworm (Lepidoptera: Noctuidae) to Cry1A.105 and Cry2Ab2 in North and South Carolina. J. Econ. Entomol..

[21] Kaur G (2019). Field-evolved resistance of Helicoverpa zea (Boddie) to transgenic maize expressing pyramided Cry1A. 105/Cry2Ab2 proteins in northeast Louisiana, the United States. J. Invertebr. Patho.

[22] Adang, M., Crickmore, N. & Jurat-Fuentes, J. L. Diversity of Bacillus thuringiensis crystal toxins and mechanism of action’, In Dhadialla, T. S. & Gill, S. (Eds.) Advances in Insect Physiology Vol. 47: Insect Midgut and Insecticidal Proteins. San Diego, CA: Academic Press, 39–87 (2014).

[23] Chakroun M, Banyuls N, Bel Y, Escriche B, Ferré J (2016). Bacterial vegetative insecticidal proteins (VIP) from entomopathogenic bacteria. Microbiol. Mol. Biol. Rev..

[24] Burkness EC, Dively G, Patton T, Morey AC, Hutchison WD (2010). Novel Vip3A Bacillus thuringiensis (Bt) maize approaches high-dose efficacy against Helicoverpa zea (Lepidoptera: Noctuidae) under field conditions. GM. Crop..

[25] Yang F (2015). Performance of Agrisure Viptera 3111 corn against Helicoverpa zea (Lepidoptera: Noctuidae) in seed mixed plantings. Crop. Prot..

[26] Andow DA, Alstad DN (1998). The F2 screen for rare resistance alleles. J. Econ. Entomol..

[27] Andow DA, Alstad DN (1999). Credibility interval for rare resistance allele frequencies. J. Econ. Entomol..

[28] Burd AD, Gould F, Bradley JR, Van Duyn JW, Moar WJ (2003). Estimated frequency of nonrecessive Bt resistance genes in bollworm, Helicoverpa zea (Boddie) (Lepidoptera: Noctuidae) in eastern North Carolina. J. Econ. Entomol..

[29] Jones RL, Perkins WD, Sparks AN (1979). Effects of sex ratios on reproduction by the corn earworm in the laboratory. Ann. Entomol. Soc. Am..

[30] Carpenter JE, Sparks AN, Pair SD, Cromroy HL (1989). Heliothis zea (Lepidoptera: Noctuidae): Effects of radiation and inherited sterility on mating competitiveness. J. Econ. Entomol..

[31] Blanco CA, Sumerford DV, Lopez JD, Hernandez G (2006). Mating incidence of feral Heliothis virescens (Lepidoptera: Noctuidae) males confined with laboratory females. J. Cotton Sci..

[32] Liu J (2007). Identification of vip3A‐type genes from Bacillus thuringiensis strains and characterization of a novel vip3A‐type gene. Lett. Appl. Microbiol..

[33] Crespo AL (2008). Comparison and validation of methods to quantify Cry1Ab toxin from Bacillus thuringiensis for standardization of insect bioassays. Appl. Env. Microbiol..

[34] Abbott WS (1925). A method of computing the effectiveness of an insecticide. J. Econ. Entomol..

[35] SAS Institute, SAS/ STAT User’s Third Edition, SAS Institute Inc, Cary, NC, USA (2010).

[36] Stodola TJ, Andow DA (2004). F2 screen variations and associated statistics. J. Econ. Entomol..

[37] Bernardi O (2015). Frequency of resistance to Vip3Aa20 toxin from Bacillus thuringiensis in Spodoptera frugiperda (Lepidoptera: Noctuidae) populations in Brazil. Crop. Prot..

[38] Bernardi, O. *et al*. Selection and characterization of resistance to the Vip3Aa20 protein from Bacillus thuringiensis in Spodoptera frugiperda. *Pest Manag Sci*, 10.1002/ps.4223 (2016). 10.1002/ps.4223 26733182

[39] Yang F (2018). F2 screen, inheritance and cross‐resistance of field‐derived Vip3A resistance in Spodoptera frugiperda (Lepidoptera: Noctuidae) collected from Louisiana, USA. Pest. Manag. Sci..

[40] Yang F, Williams J, Porter P, Huang F, Kerns DL (2019). F2 screen for resistance to Bacillus thuringiensis Vip3Aa51 protein in field populations of Spodoptera frugiperda (Lepidoptera: Noctuidae) from Texas, USA. Crop. Prot..

[41] Yang F (2013). Susceptibility of Louisiana and Florida populations of Spodoptera frugiperda (Lepidoptera: Noctuidae) to transgenic Agrisure Viptera 3111 corn. Crop. Prot..

[42] Mahon RJ, Downes SJ, James B (2012). Vip3A resistance alleles exist at high levels in Australian targets before release of cotton expressing this toxin. PLoS One.

[43] Hendricks DE, Graham HM, Fernandez AT (1970). Mating of female tobacco budworms and bollworms collected from light traps. J. Econ. Entomol..

[44] Lamunyon CW (2000). Sperm storage by females of the polyandrous noctuid moth Heliothis virescens. Anim. Behav..

[45] Blanco CA, Sumerford DV, Lopez JD, Hernandez G, Abel CA (2010). Mating behavior of wild Helicoverpa zea (Lepidoptera: Noctuidae) males with laboratory females. J. Cotton Sci..

[46] Tabashnik BE (1994). Evolution of resistance to Bacillus thuringiensis. Annu. Rev. Entomol..

[47] Raymond B, Sayyed AH, Wright DJ (2005). Genes and environment interact to determine the fitness costs of resistance to Bacillus thuringiensis. Proc. R. Soc. Lond. B Biol. Sci..

[48] Gassmann AJ, Stock SP, Carrière Y, Tabashnik BE (2006). Effect of entomopathogenic nematodes on the fitness cost of resistance to Bt toxin Cry1Ac in pink bollworm (Lepidoptera: Gelechiidae). J. Econ. Entomol..

[49] Bird LJ, Akhurst RJ (2007). Effects of host plant species on fitness costs of Bt resistance in Helicoverpa armigera (Lepidoptera: Noctuidae). Biol. Control..

[50] Wang R, Tetreau G, Wang P (2016). Effect of crop plants on fitness costs associated with resistance to Bacillus thuringiensis toxins Cry1Ac and Cry2Ab in cabbage loopers. Sci. Rep..

[51] Chen X (2019). Fitness costs of Vip3A resistance in Spodoptera frugiperda on different hosts. Pest. Manag. Sci..

